# Economic impact of switching from partially combined vaccine “Pentaxim® and hepatitis B” to fully combined vaccine “Hexaxim®” in the Malaysian National Immunization Program

**DOI:** 10.1186/s12913-021-07428-7

**Published:** 2022-01-05

**Authors:** Syed Mohamed Aljunid, Lama Al Bashir, Aniza Binti Ismail, Azimatun Noor Aizuddin, S. A. Zafirah Abdul Rashid, Amrizal Muhammad Nur

**Affiliations:** 1 International Centre for Casemix and Clinical Coding, Hospital Canselor Tuanku Muhriz, National University of Malaysia, Kuala Lumpur, Malaysia; 2 Department of Health Policy and Management, Faculty of Public Health, Kuwait University, Kuwait City, Kuwait; 3 Malaysian Health Economic Association (MAHEA), International Centre for Casemix and Clinical Coding, Hospital Canselor Tuanku Muhriz, National University of Malaysia, Kuala Lumpur, Malaysia; 4 Department of Community Health, Faculty of Medicine, University Kebangsaan Malaysia, Bangi, Selangor Malaysia; 5 Casemix Solutions Sdn Bhd, International Centre for Casemix and Clinical Coding,Hospital Canselor Tuanku Muhriz, National University of Malaysia, Kuala Lumpur, Malaysia

**Keywords:** Economic impact, Hexaxim, Pentaxim, Malaysia national immunization program

## Abstract

**Background:**

The decision to implement new vaccines should be supported by public health and economic evaluations. Therefore, this study was primarily designed to evaluate the economic impact of switching from partially combined vaccine (Pentaxim® plus hepatitis B) to fully combined vaccine (Hexaxim®) in the Malaysian National Immunization Program (NIP) and to investigate healthcare professionals (HCPs)’ and parents’/caregivers’ perceptions.

**Methods:**

In this economic evaluation study, 22 primary healthcare centers were randomly selected in Malaysia between December 2019 and July 2020. The baseline immunization schedule includes switching from Pentaxim® (four doses) and hepatitis B (three doses) to Hexaxim® (four doses), whereas the alternative scheme includes switching from Pentaxim® (four doses) and hepatitis B (three doses) to Hexaxim® (four doses) and hepatitis B (one dose) administered at birth. Direct medical costs were extracted using a costing questionnaire and an observational time and motion chart. Direct non-medical (cost for transportation) and indirect costs (loss of productivity) were derived from parents’/caregivers’ questionnaire. Also, HCPs’ and parent’s/caregivers’ perceptions were investigated using structured questionnaires.

**Results:**

The cost per dose of Pentaxim® plus hepatitis B vs. Hexaxim® for the baseline scheme was Malaysian ringgit (RM) 31.90 (7.7 United States dollar [USD]) vs. 17.10 (4.1 USD) for direct medical cost, RM 54.40 (13.1 USD) vs. RM 27.20 (6.6 USD) for direct non-medical cost, RM 221.33 (53.3 USD) vs. RM 110.66 (26.7 USD) for indirect cost, and RM 307.63 (74.2 USD) vs. RM 155.00 (37.4 USD) for societal (total) cost. A similar trend was observed for the alternative scheme. Compared with Pentaxim® plus hepatitis B, total cost savings per dose of Hexaxim® were RM 137.20 (33.1 USD) and RM 104.70 (25.2 USD) in the baseline and alternative scheme, respectively. Eighty-four percent of physicians and 95% of nurses supported the use of Hexaxim® in the NIP. The majority of parents/caregivers had a positive perception regarding Hexaxim® vaccine in various aspects.

**Conclusions:**

Incorporation of Hexaxim® within Malaysian NIP is highly recommended because the use of Hexaxim® has demonstrated substantial direct and indirect cost savings for healthcare providers and parents/caregivers with a high percentage of positive perceptions, compared with Pentaxim® plus hepatitis B.

**Trial registration:**

Not applicable.

**Supplementary Information:**

The online version contains supplementary material available at 10.1186/s12913-021-07428-7.

## Background

Childhood vaccination programs have been a vital public health intervention that reduced the incidence of vaccine preventable diseases by more than 95% for every pediatric vaccine recommended in the immunization schedules [1]. In Malaysia, the immunization schedule included administration of hepatitis B (Hep B) vaccine at birth and 1 and 6 months and of Pentaxim® (combination vaccines) at 2, 3, 5, and 18 months. Pentaxim® is a pentavalent vaccine that confers protection against the following five infectious diseases: diphtheria, tetanus, pertussis, poliomyelitis, and *Haemophilus influenzae* type b (Hib). It is administered as a pre-filled liquid syringe that contains diphtheria, tetanus, acellular pertussis combined with inactivated polio vaccine (DTaP-IPV) and must be mixed or reconstituted with Hib vial (white lyophilized powder) immediately before injection. Nevertheless, even though the National Immunization Program (NIP) incorporates many combination vaccines, the immunization schedule is still somehow crowded and complex. This situation will worsen in the future as new diseases emerge and/or new vaccines are developed [2]. Therefore, development of new combination vaccines that can simplify the vaccination schedule safely and efficiently is the unmet need [3].

The combined DTaP vaccine has already been incorporated into the national immunization schedules in most countries [4]. These DTaP-based vaccines serve as the backbone of several other combination vaccines, such as tetravalent, pentavalent, and hexavalent vaccines [5]. The choice of using combination vaccine over monovalent vaccines in the childhood immunization schedule was proofed by several previous research, wherein combined vaccine seroprotection (i.e., the percentages of infants with antibody titers above predefined protective levels for anti-diphtheria; −tetanus; −polio 1, 2, and 3; and -Hib vaccine [polyribosylribitol phosphate]) and seroconversion rates (i.e., the percentages of infants showing a four-fold increase in pertussis antigens [anti-pertussis toxin and anti-filamentous hemagglutinin adhesin] were tested [5–16]. These clinical trials showed the immunogenicity of a primary series 1 month after the three-dose primary vaccination with combined vaccine (Pentaxim); 92.2 to 100% of infants achieved seroprotective levels of anti-diphtheria, anti-tetanus, anti-polio types 1, 2, and 3, and anti-PRP. The seroconversion rate was 83.9–100% for pertussis antigens (anti-pertussis toxin and anti-filamentous hemagglutinin adhesin). Combined vaccine-generated antibody responses were comparable to those following separately administered vaccines, including DTaP, oral polio vaccine, IPV, and Hib vaccine [17].

Recently, a new, fully liquid, ready-to-use hexavalent DTaP-IPV-Hep B-Hib combination vaccine (Hexaxim®, Sanofi Pasteur) has been developed and administered at 2, 3, 5, and 18 months of age, with no reconstitution needed prior to administration [18]. Hexaxim® vaccine was approved by the European Medicine Agency and the World Health Organization in 2015. The approval of the DTaP-Hep B-IPV-Hib vaccine was based on the review of European Medicine Agency/World Health Organization reports related to the quality, safety, and efficacy data [19]. In 2019, it was used as the only hexavalent brand in the national immunization schedule in the following 12 countries: South Africa, Saudi Arabia, Mexico, Iraq, Libya, Chile, Panama, Kazakhstan, Belgium, Austria, Norway, and Georgia [4]. As a matter of fact, in 2019, Hexaxim® held the leading position in the hexavalent vaccine market with 62% (approximately 22 million doses) of the volumes consumed across the globe, and it is the most used hexavalent vaccine in public markets with 69% of market share [4]. In addition, an analysis of vaccinations for pre-term infants (babies born alive before 37 weeks of pregnancy) showed that Hexaxim® was the most used pre-term hexavalent vaccine, with a volume share of 66% (approximately 1.7 million doses), and was used across 16 countries (Argentina, Austria, Belgium, Brazil, Chile, Croatia, Georgia, Iraq, Kazakhstan, Libya, Macedonia, Mexico, Norway, Panama, Saudi Arabia, and South Africa) [4].

A ready-to-use combination vaccine can reduce costs, simplify logistics and delivery infrastructure, and improve coverage with fewer injections [20]. Madhi et al. demonstrated that a fully liquid, hexavalent DTaP-IPV-Hep B-Hib vaccine (Hexaxim®) is highly immunogenic and safe in South African infants under the Expanded Programme on Immunization [21]. In addition, similar safety profiles of Pentaxim® plus monovalent Hep B and Hexaxim® vaccine were found in Korean infants [22]. Mogale et al. recommended implementation of Hexaxim® within the South African Expanded Programme on Immunization because it reduces healthcare providers’ cost [20].

Based on the above-mentioned facts, Hexaxim® vaccine appears to be a better choice to replace Pentaxim® plus Hep B in the Malaysian NIP, especially within the context of growing financial burden on the Malaysian healthcare system. In Malaysia, Hexaxim® is currently in use in the private medical sector, but Pentaxim is still in use in the public medical sector. Nevertheless, in 2019, the Ministry of Health (MOH), Malaysia has launched a pilot project on the implementation of Hexaxim® in the Malaysian NIP across public health centers (PHCs) located in Selangor, Federal Territory of Kuala Lumpur and Putrajaya. To date, the adoption of Hexaxim® in the Malaysia NIP is still inconclusive. Since the decision to implement new vaccines should be supported by public health and economic evaluations that are becoming increasingly important for policymakers, this study was primarily designed to estimate the economic impact (direct medical costs, direct non-medical costs, and indirect costs) of using Hexaxim® vaccine compared with Pentaxim® plus Hep B vaccines in the Malaysian NIP and to investigate healthcare professionals (HCPs)’ and parents’/caregivers’ perceptions.

## Methods

### Study design and participants

In this study, three types of study design namely economic evaluation, observational time and motion, and cross-sectional study were employed to achieve the study objectives. A total of 22 primary healthcare centers (PHCs), which provide childhood vaccination services, were selected using stratified sampling method from the states of Selangor (18 PHCs) and the federal territory of Kuala Lumpur and Putrajaya state (4 PHCs). These clinics were selected due to their involvement in the pilot project of Hexaxim® vaccine. The study was conducted between December 2019 and July 2020. The following study participants were included in this study: (1) physicians and nurses who had at least 1 year of experience in the field of childhood vaccinations and were in charge of counseling or prescribing, administering and/or reconstituting Pentaxim® and Hep B injections, or supervising the vaccination process in PHCs and (2) parents (aged ≥18 years) of infants/toddlers who aged between 1 and 24 months.

The study was approved by the National University of Malaysia Research and Ethics Committee (ethics committee approval reference # FF-2019-318) and the Medical Research and Ethics Committee of the Ministry of Health Malaysia (ethics committee approval reference # NMRR-19-1370-47,862). The study was performed according to the local and national regulations. Also, it was consistent with the standards established by the Declaration of Helsinki and compliant with the International Council for Harmonization guidelines for Good Clinical Practice. An informed consent form was signed by each participant’s parent(s) or legally acceptable representative(s) before enrollment into each study. If the parents were illiterate, an independent witness fully explained and signed the informed consent form.

### Outcome measures and data collection

#### Economic evaluation

The economic evaluation study was conducted using a costing questionnaire, time and motion chart, and parents’/caregivers’ questionnaire. The economic evaluation was based upon two immunization schemes: (1) baseline scheme – the immunization schedule had a transition from four doses of Pentaxim® and three doses of Hep B to only four doses of Hexaxim® and (2) alternative scheme – it involves moving from Pentaxim® (four doses) and Hep B (three doses) to Hexaxim® (four doses) and one Hep B dose administered at birth. An economic model was prepared by the study authors to set the proper formula used to calculate the cost of direct medical, direct non-medical, and indirect cost. Then it was validated and approved by a panel of experts in the field of family medicine, public health, and epidemiology and representatives from the MOH in Malaysia.

##### (a) Costing questionnaire

The costing questionnaire had two versions: one directed to the PHCs (Supplementary Table 1) and the other to the district health office (Supplementary Table 2). The costing questionnaire was developed upon reviewing previous related studies [20, 23, 24] and modified after several field visits to the PHCs to evaluate all the supplies and expenses included in the vaccination process. One part of the costing questionnaire was directed to the matron or head nurse of maternal and child department, and the other part was directed to the related staff in the district health office. The costing questionnaire was designed to extract raw data, transformed to direct medical cost of vaccines borne by the healthcare provider. This cost included cost of consumables (swabs, syringes, needles, and safety box), hazardous waste disposal, vaccine wastage, and cold chain storage (refrigerator and cold box).

##### (b) Time and motion chart

The time and motion chart (Supplementary Table 3) was designed to record the time spent by 46 nurses to accomplish tasks associated with vaccination process; this recorded time was used to calculate the cost of vaccine administration time, which is a component of direct medical cost borne by the health care provider. Time and motion chart was prepared after reviewing previous related studies [20, 25]. It was modified after several field visits to the PHCs to entail all the steps included in the vaccination visits and undertaken all the detailed steps in the injection process. This chart was reviewed and validated by the head nurse of each PHCs visited to ensure they follow the same ordered steps in the vaccination process.

##### (c) Parents’/caregivers’ questionnaire

The parents’/caregivers’ questionnaire (Supplementary Table 4) was used to extract data that were used to calculate direct non-medical and indirect costs. The cost of the vaccine borne by the parents/caregivers includes the cost for transportation (direct non-medical cost) and loss of productivity (indirect cost).

### Perception’s assessment

This cross-sectional study of perceptions was conducted using a structured and validated questionnaire of parents’/caregivers’ perceptions (Supplementary Table 4) and that of healthcare practitioners’ perceptions (Supplementary Tables 5 and 6), where a five-point Likert scale (Strongly Disagree, Agree, Neutral, Disagree, and Strongly Disagree) was employed in the questionnaires [26, 27].

A total of 350 parents/caregivers were chosen for parents/caregivers based on a previous perception’s study conducted in Malaysia, by Ahmad et al. in 2017 [28], and 150 HCPs were recruited based on a study by Bakhache et al. in 2013 [29].

The questionnaire’s content validity was tested through a focus group discussion attended by family medicine physician, pediatrician, and public health medicine specialist. After the content was validated by the experts, 10% of the sample was selected for face validity. These samples were then excluded during the study, and the questionnaire was amended according to the finding of the face validity. The construct validity of the questionnaire was validated through the pilot study. Then, the data collected during the pilot study were used to perform a factor analysis. Reliability and validity of parents’/caregivers’ perceptions questionnaire were finally confirmed by the Cronbach’s alpha of 0.91 and Kaiser–Meyer–Olkin’s value of 0.88 (*p* < 0.001). The Cronbach’s alpha for nurses’ and physicians’ perceptions questionnaire ranged from 0.87 to 0.92, and the validity was confirmed by Kaiser–Meyer–Olkin’s value of 0.69 (*p* < 0.001) and 0.74 (*p* < 0.001) for nurses’ and physicians’ perceptions questionnaire, respectively.

The parents’ questionnaire was distributed among 346 parents of vaccinated children, who were chosen by systematic sampling, provided information about their sociodemographic characteristics, and expressed their opinion and perceptions regarding the employment of Hexaxim® vaccine in the immunization schedule. Healthcare practitioners’ questionnaire had two versions, one directed toward nurses and the other toward physicians who are working in the maternal and child health (MCH) units in PHCs where both were chosen by either universal or systematic sampling depending upon the HCP working in that MCH unit. Nurses’ version was collected from 100 nurses who provided information about their sociodemographic and clinical practice profile and conveyed their perceptions regarding Pentaxim® vaccine and about switching to fully combined vaccine. Moreover, 50 physicians participated in the physicians’ perceptions questionnaire and answered the questions regarding their perception of incorporating Hexaxim® vaccine in the NIP transparently and vividly.

### Statistical analyses

The costing method employed in the economic evaluation study was based upon the societal perspective, that is, the sum of costs paid by providers and parents/caregivers. The vaccine societal cost was calculated as cost per dose, per fully immunized child (FIC), and per birth cohort, and then the net cost savings were calculated for each. There are 11 components of the cost analysis. Table 1 below summarizes the cost components and the formula. The specific formula and inputs for each cost component are summarized in Supplementary Table 7.

**Table 1:** Cost components

Cost component	Formula
**Swab**	Number of swabs used per dose of vaccine × swab price per piece
**Syringe**	Number of syringes per dose of vaccine × price of one syringe × syringes wastage factor
**Needle**	Number of needles per dose of vaccine × price of one needle × needles wastage factor
**Safety box**	(Syringe volume × no. of syringes per dose of vaccine) + (needle volume × no. of needles per dose of vaccine) + (vaccine vial volume × no. of vials per dose of vaccine) × (price of empty safety box)/actual safety box volume (mL) (2/3 of safety box gross volume)
**Hazardous waste disposal**	(Empty syringe weight × no. of syringes disposed per dose of vaccine) + needle weight × no. of needles per dose of vaccine) + (empty vaccine vial weight × no. of empty vials per dose of vaccine)/1000 × hazardous waste disposal price per 1000 g
**Vaccine wastage**	Purchase price of vaccine per dose × vaccine wastage factor
**Cold chain: refrigerator**	Vaccine package volume per dose (mL) × (refrigerator electricity expenditure per day × electricity price per kWh/refrigerator vaccine storage capacity (mL))
**Cold chain: cold box**	(Refrigerator or freezer electricity expenditure kWh × electricity price per kWh/freezer gross volume (mL)) × (ice pack total volume per cold box × vaccine volume per dose/cold box volume (mL))
**Administration time**	Monthly specific nurse salary (basic salary)/working hours per month/3600 × time (s) to administer one dose of vaccine
**Transportation**	Cost of transportation caregivers have to pay to and from the primary healthcare center per visit
**Loss of productivity**	Caregiver’s monthly salary/26 × time absent from work due to vaccination (days)

Sensitivity analyses were conducted to investigate how sensitive the findings of an economic evaluation are to changes in the assumptions used in the study and to examine variations in the parameter estimates. Sensitivity analyses were calculated based on vaccine wastage rate (2.5 and 10%) and administration time (minimum and maximum) as both are the key variables that affect the cost saving.

All the data for the direct medical cost were collected by the principal authors. On the other hand, the direct non-medical and indirect costs were collected by a team of four research assistants. These research assistants are newly graduated physicians and have been trained by the principal authors regarding the study scope and the questionnaires. Data were analyzed using Excel Microsoft Office spreadsheet (version 2013) and SPSS (version 22) where descriptive tables with frequencies and percentages for all the categorical variables were generated. Mean and standard deviation were calculated for all continuous variables.

## Results

### Economic evaluation

A summary of cost borne by providers (direct medical), parents (direct non-medical and indirect cost), and societal (total) vaccine cost by immunization schemes is shown in Tables 2, 3, and 4, respectively. In the baseline scheme, the cost per dose of partially combined vaccine vs. Hexaxim® was Malaysian ringgit (RM) 31.90 vs. 17.10 (direct medical cost), RM 54.40 vs. RM 27.20 (direct non-medical cost), RM 221.33 vs. RM 110.66 (indirect cost), and RM 307.63 vs. RM 155.00 (societal cost). Cost savings with Hexaxim® were as follows: RM 137.20 per dose, RM 458.00 per FIC, and RM 267,977,435 per birth cohort. In the alternative scheme, cost per dose for both partially and fully vaccines was the same as in the baseline scheme since the additional vaccine effect was added to the cost per FIC. The cost per FIC of partially combined vaccine vs. Hexaxim® was RM 112.91 vs. 83.10 (direct medical cost), RM 190.40 vs. RM 136.00 (direct non-medical cost), and RM 774.64 vs. RM 553.31 (indirect cost). Cost savings with Hexaxim® were as follows: RM 104.70 per dose, RM 305.50 per FIC, and RM 204,540,947 per birth cohort.

**Table 2:** Summary of cost borne by provider (direct medical) by immunization schemes

Cost components		Cost (RM) per dose of vaccine					
		Baseline scheme^a^			Alternative scheme^b^		
		Pentaxim	Hepatitis B	Hexaxim®	Pentaxim	Hepatitis B	Hexaxim®
**1.a**	Swabs	0.03583	0.03583	0.03583	0.03583	0.03583	0.03583
**1.b**	Syringe	0.32579	0.32579	0.32579	0.32579	0.32579	0.32579
**1.c**	Needles	0.18537	0.18537	0.18537	0.18537	0.18537	0.18537
**1.d**	Safety box	0.00109	0.00073	0.00073	0.00109	0.00073	0.00073
**2**	Hazardous waste disposal	0.15000	0.10000	0.10000	0.15000	0.10000	0.10000
**3**	Vaccine wastage^c^	2.60450	0.21460	2.60450	2.60450	0.21460	2.60450
**4.a**	Refrigerator	0.00212	0.00020	0.00016	0.00212	0.00020	0.00016
**4.b**	Cold box	0.00056	0.00001	0.00001	0.00056	0.00001	0.00001
**5**	Administration time	13.91216	13.82024	13.81065	13.91216	13.82024	13.81065
**Total per dose**		17.21742	14.68276	17.06303	17.21742	14.68276	17.06303
**Total per FIC**		68.86968	44.04828	68.25211	68.86968	44.04827941	68.25211
**Comparison of cost (RM)**		**Pentaxim® + hepatitis B**	**Hexaxim®**		**Pentaxim® + hepatitis B**	**Hexaxim®**	
Cost per dose		31.90	17.10		31.90	17.10	
Cost per FIC		112.91	68.40		112.90	83.10	
Cost savings per FIC		44.50			29.80		
Cost per birth cohort (2019)^d^		55,142,582	33,402,593		55,142,582	40,581,220	
Cost savings per birth cohort		21,739,989			14,561,362		
Cost savings per dose		11.10			7.50		

*Abbreviations*: *FIC* fully immunized child, *RM* Malaysian ringgit

^a^Based on four doses of Hexaxim®

^b^Per four doses of Hexaxim® + 1 dose at birth for hepatitis B

^c^Vaccine wastage rate: 5%, according to the WHO for single dose vaccines

^d^Birth cohort (2019): 488,342 live birth according to the Department of Statistics, Malaysia

**Table 3:** Summary of cost borne by parents (direct non-medical and indirect cost) by immunization schemes

Comparison of cost saving (RM)	Perspective	Baseline scheme^a^		Alternative scheme^b^	
		Pentaxim® + hepatitis B	Hexaxim®	Pentaxim® + hepatitis B	Hexaxim®
Cost per dose	Transportation	54.40	27.20	54.40	27.20
	Loss of productivity	221.33	110.66	221.33	110.66
	Total	275.73	137.86	275.73	137.86
Cost per FIC	Transportation	190.40	108.80	190.40	136.00
	Loss of productivity	774.64	442.65	774.64	553.31
	Total	965.04	551.45	965.04	689.31
	Saving	413.60		275.73	
Cost per birth cohort (2019)^c^	Transportation	92,980,317	53,131,610	92,980,317	66,414,512
	Loss of productivity	378,288,574	171,899,835	378,288,574	214,874,794
	Total	471,268,891	225,031,445	471,268,891	281,289,306
	Saving	246,237,446		189, 979,585	
Cost savings per dose of Hexaxim®		126.06		97.26	

*Abbreviations*: *FIC* fully immunized child, *RM* Malaysian ringgit

^a^Based on four doses of Hexaxim®

^b^Per four doses of Hexaxim® + one dose at birth for hepatitis B

^c^Birth cohort (2019): 488,342 live birth according to the department of statistics Malaysia

**Table 4:** Summary of societal (total) vaccine cost by immunization schemes

Comparison of cost savings (RM)	Cost perspective	Baseline scheme^a^		Alternative Scheme 1^b^	
		Pentaxim® + hepatitis B	Hexaxim®	Pentaxim® + hepatitis B	Hexaxim®
Cost per dose	Direct medical	31.90	17.10	31.90	17.10
	Direct non-medical	54.40	27.20	54.40	27.20
	Indirect	221.33	110.66	221.33	110.66
	Societal	307.63	155.00	307.63	155.00
Cost per FIC	Direct medical	112.91	68.40	112.91	82.93
	Direct non-medical	190.40	108.80	190.40	136.00
	Indirect	774.64	442.65	774.64	553.31
	Societal	1077.95	619.90	1077.95	772.40
	Saving	458.00		305.50	
Cost per birth cohort (2019)^c^	Direct medical	55,142,582	33,402,593	55,142,582	40,581,220
	Direct non-medical	92,980,317	53,131,610	92,980,317	66,414,512
	Indirect	378,288,574	171,899,835	378,288,574	214,874,794
	Societal	526,411,473	258,434,038	526,411,473	321,870,526
	Saving	267,977,435		204,540,947	
Cost savings per dose of Hexaxim®		137.20		104.70	

*Abbreviations*: *FIC* fully immunized child, *RM* Malaysian ringgit

^a^Based on four doses of Hexaxim®

^b^Per four doses of Hexaxim® + 1 dose at birth for hepatitis B

^c^Birth cohort (2019): 488,342 live birth according to the Department of Statistics, Malaysia

**Fig. 1:**
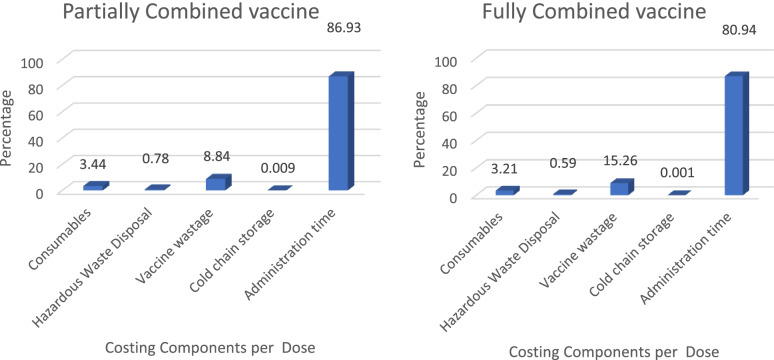
Direct medical cost profile for the baseline scheme. The relative contribution of cost components to the total cost per dose for partially combined vaccine (Pentaxim® + hepatitis B) vs. fully combined vaccine (Hexaxim®)

Figure 1 demonstrates the percentages of cost components contribution in the direct medical vaccine cost per dose for the baseline scheme. The major contributor to direct medical cost per dose was the cost of administration time (86.9% for the partially combined vaccine vs. 80.9% for fully combined vaccine), followed by the cost of vaccine wastage per dose (8.85 vs. 15.2% for partially and fully combined vaccines respectively).

Sensitivity analysis of direct medical cost (Table 5) for the baseline scheme shows that cost saving per birth cohort for the first scenario was RM 20,798,226 and that per dose of Hexaxim® was RM 10.65. For the second scenario, cost saving per birth cohort was RM 24,219,354 and that per dose of Hexaxim® was RM 12.4. Sensitivity analysis of the alternative scheme (Table 5) shows that for the first scenario, cost saving per birth cohort was RM 13,914,263 and that per dose of Hexaxim® was RM 7.12. For the second scenario, cost saving per birth cohort was RM 16,307,093 and that per dose of Hexaxim® was RM 8.35. The results of sensitivity analysis are consistent with the results of the primary analysis, indicating that incorporating Hexaxim® in the NIP will yield direct medical cost savings in both immunization schemes, irrespective of the variation in the assumptions used for the cost calculations (i.e., wastage rate and administration time).

**Table 5:** Sensitivity analysis of direct medical cost by immunization schemes

Cost components	Cost (RM) per dose of vaccine					
	Baseline scheme^a^			Alternative scheme^b^		
	Pentaxim® + hepatitis B		Hexaxim®	Pentaxim® + hepatitis B		Hexaxim®
**Scenario 1**^d^						
Cost per dose	29.48		15.31	29.48		15.31
Cost per FIC	103.84		61.25	103.84		75.35
Cost per birth cohort (2019)	50,711,216		29,912,990	50,711,216		36,796,952
**Scenario 2**^e^						
Cost per dose	37.62		21.17	37.62		21.17
Cost per FIC	134.29		84.70	134.29		100.90
Cost per birth cohort (2019)	65,584,301		41,364,947	65,584,301		49,277,207
**Comparison of cost savings (RM)**	**Baseline scheme**			**Alternative scheme**		
	**Baseline case**^c^	**Scenario 1**^d^	**Scenario 2**^e^	**Baseline case**^c^	**Scenario 1**^d^	**Scenario 2**^e^
Cost savings per FIC	44.7	42.59	49.60	29.98	28.49	33.39
Cost savings per birth cohort	21,812,210	20,798,226	24,219,354	14,642,002	13,914,263	16,307,093
Cost savings per dose of Hexaxim®	11.1	10.56	12.40	7.50	7.12	8.35

*Abbreviations*: *FIC* fully immunized child, *RM* Malaysian ringgit

^a^Based on four doses of Hexaxim®

^b^Per four doses of Hexaxim® + 1 dose at birth for hepatitis B

^c^Baseline: Wastage rate 5%, mean administration time

^d^Scenario 1: Wastage rate 2.5%, minimum administration time (Pentaxim® + hepatitis B: 98.2 min; Hexaxim®: 48.56 min)

^e^Scenario 2: Wastage rate 10%, maximum administration time (Pentaxim® + hepatitis B: 112.23 min; Hexaxim®: 55.29 min)

### Perceptions assessment

A summary of parents/caregivers’ and HCPs’ perceptions is presented in Table 6. A total of 346 parents/caregivers (mean age: 31.90 years [range: 19–59 years], 70.5% female) participated in the perception study. Majority of parents/caregivers had positive perceptions toward Hexaxim® vaccine, where 94.2% of them thought that Hexaxim® usage would reduce their child’s pain and discomfort due to less injection administered compared with the current immunization schedule. In addition, 99.1, 97.1, and 95.6% of parents/caregivers agreed that Hexaxim® could reduce their visits to PHCs, decrease their transportation expenses to reach PHCs, and increase their compliance to the vaccination schedule, respectively. Moreover, 97.1% of parents/caregivers supported the idea of reviewing the current schedule and incorporating Hexaxim® in it. One hundred nurses (mean age: 34.85 years and mean experience in childhood vaccination: 8.39 years) completed the perceptions questionnaire. In all, 70.0, 78.0, 79.0, and 74.0% of nurses believed that Pentaxim® vaccine reconstitution is a time loss, requires too many steps compared with other vaccines, could lead to handling errors, and has a greater chance of needlestick injury, respectively. On the other side, 91.0 and 87.0% of nurses agreed that Hexaxim® usage could reduce their daily work burden and patient influx (arrival) to the PHCs, respectively. This could be the reason 95.0% of nurses supported Hexaxim® employment in the NIP. A total of 50 physicians (mean age: 32.70 years and average experience in childhood vaccination: 4.47 years) participated in the study. In all, 80.0% of physicians thought that Hexaxim® replacement instead of Pentaxim® and Hep B could yield cost savings to the provider or government. In all, 60.0% of physicians believed that parents would support Hexaxim® incorporation, and 84.0% of physicians believed that Hexaxim® incorporation would increase their compliance. Also, 82.0% of physicians agreed that Hexaxim® could reduce the daily patient influx and crowdedness at PHCs. In addition, 82.0% of physicians also thought that Hexaxim® would ease the incorporation of pneumococcal conjugate vaccine into the immunization schedule. Lastly, 84.0% of physicians supported Hexaxim® employment in the Malaysian NIP.

**Table 6:** Summary of parents’/caregivers’ and healthcare professionals’ perceptions

Perception item	Agree (%) (strongly agree or agree)	Neutral (%)	Disagree (%) (strongly disagree or disagree)
**Parents’/Caregivers’ perception**			
Hexaxim® reduced pain and discomfort	94.20	5.20	0.60
Hexaxim® reduce number of visits	99.10	0.90	0.00
Hexaxim® reduce transportation expenses	97.10	2.90	0.00
Hexaxim® increase compliance	95.60	4.10	0.30
Current immunization schedule Should be reviewed	97.10	2.30	0.60
**Nurses’ perceptions**			
Pentaxim® cause time lost	70.00	17.00	13.00
Pentaxim® has too many steps in reconstitution	78.00	11.00	11.00
Pentaxim® may lead to handling errors	79.00	6.00	15.00
Pentaxim® may cause more needle stick injury	74.00	9.00	17.00
Hexaxim® can reduce staff workload	91.00	7.00	2.00
Hexaxim® can reduce patient overcrowding in clinics	87.00	9.00	4.00
Support Hexaxim® employment	95.00	3.00	2.00
**Physicians’ perceptions**			
Support Hexaxim® employment	84.00	6.00	10.00
Parents interested to replace Pentaxim® with Hexaxim®	60.00	32.00	8.00
Hexaxim® leads to cost saving	80.00	20.00	0.00
Hexaxim® can reduce patient overcrowding in clinics	82.00	10.00	8.00
Hexaxim® can ease incorporation of other vaccines, e.g., pneumococcal conjugate vaccine (PCV)	82.00	18.00	0.00
Hexaxim® may enhance compliance to immunization schedule	84.00	8.00	8.00

## Discussion

Combination vaccines are promoted to overcome the problems associated with multiple administration of monovalent vaccines. The use of combination vaccines, which include several antigens in a single administration, offers benefits such as reduced complications associated with multiple intramuscular injections, decreased costs of stocking and administering separate vaccines, and a lowering of the risk of delayed or missed vaccinations. Therefore, the development of combined vaccines has been a public health interest and a priority, and their use is recommended by the WHO [23, 24]. To the best of our knowledge, this study is the first to evaluate the cost implications and HCPs’/parents’ perception of a switch from the current combination of Pentaxim® plus Hep B injections to a single Hexaxim® injection in the Malaysian NIP.

The economic evaluation results demonstrated that Hexaxim® had a lower cost per dose, per FIC, and per birth cohort (2019) and significant cost savings with regards to direct medical cost borne by HCP and direct non-medical cost (transportation) and indirect cost (loss of productivity) borne by parents/caregivers, compared with Pentaxim® plus Hep B. These results are supported by the results of a similar cost minimization study (from the public sector perspective only) conducted in South Africa in 2014 that also analyzed replacing Pentaxim® and Hep B vaccine with Hexaxim® vaccine [20]. The direct medical cost saving per dose of Hexaxim® for the present study was RM 11.10 (in baseline scheme) compared with RM 7.4 (29.4 African rand) in the South African study [20]. Moreover, based upon the costing profile generated in this study, it appeared that administration time was the cost component that contributed the most to the total direct cost per dose of Pentaxim® plus Hep B (86.9%) and Hexaxim® (80.9%). In contrast, Mogale et al. showed that cold chain storage was the major cost component for both partially and fully combined vaccines [20]. This inconsistency arose due to the different methods used to calculate the administration cost. To assess the cost of cold chain storage, Mogale et al. have used the capital costs, which means the researchers calculated how much space each vaccine would occupy compared with the refrigerator cost or cost of appliance. This method yielded a higher cost because of the expensive purchase price of vaccine appliances. In the current study, cold chain storage cost calculation was based upon the recurrent cost of energy for each dose of the vaccine, which resulted in a relatively minor contribution to the cost per dose compared with the other cost components for partially (0.009%) and fully combined (0.0009%) vaccines.

In the present study, the majority of parents believed that using Hexaxim® vaccine could reduce their child’s pain and discomfort compared with Pentaxim® plus Hep B. This was similar to a combination perception study conducted in the United States by Petraco and Judelsohn [30], where all parents wished new childhood vaccinations to be available in a combination form, so that their infants do not have to get too many shots or injections to avert the extra pain they have to suffer. Per the Malaysian NIP, parents must make five vaccination visits during the first 6 months of their infants’ life, which increases the direct non-medical cost (transportation) and indirect cost (loss of productivity). The parents who participated in this study “Agreed” unanimously that replacing Pentaxim® and Hep B with Hexaxim® could reduce the number of visits and, consequently, the transportation expenses. Based upon that, majority of parents in this study believed that Hexaxim® incorporation in the immunization schedule could lead to more vaccination compliance. According to Hull and McIntyre [31], vaccination delays increase with number of doses or visits, where the number of immunizations and the complexity of the schedule are the primary reasons for vaccine dose deferrals and non-compliance. Most of the parents in this study demanded an immunization schedule review if there is a new vaccine, such as Hexaxim®, that can simplify the schedule.

In the present study, more than three-quarter of nurses believed that Pentaxim® vaccine reconstitution is a time loss because it requires too many steps to prepare, whereas Hexaxim® vaccine usage could reduce the work burden as, like Pentaxim®, it does not need reconstitution, which can save nurses’ time and efforts. Our finding is consistent with the result of a randomized, crossover, time and motion study conducted at Belgium by De Coster et al. [25], which reported that preparation time for non-fully liquid vaccine (70.5 s) was double than that for fully liquid vaccines (36.0 s). Furthermore, Pellissier et al. stated that time saving due to fully liquid vaccine can allow more time for patient education over a broad range of healthcare issues and increases the quality of care that HCPs can offer [32]. Moreover, more than three-quarter of nurses in the current study believed that Pentaxim® reconstitution could lead to handling errors, which is consistent with the results of the time and motion study conducted by De Coster et al., in which it was found that non-fully liquid vaccine reconstitution led to 24.48% of the immunization errors compared with 5.2% for the fully liquid vaccines [25]. In the present study, the majority of nurses supported Hexaxim® employment in the vaccination schedule, which is consistent with De Coster et al.’s study [25] in which 97.6% of HCPs participated in the study preferred the use of the fully liquid vaccine in their daily clinical practice.

In the present study, more than three-quarter of physicians believed that Hexaxim® incorporation in the Malaysian NIP can produce substantial cost savings for both healthcare providers and parents. This is supported by the high percentage of positive perceptions regarding Hexaxim® obtained from parents and healthcare providers. This high percentage of agreement is due to two reasons: (1) physicians are aware of the limited budget dedicated to the health sector and (2) all the physicians participated in the study are medical officers who often deal with the financial issues of the health center. In the present study, the physicians’ perceptions analysis indicated that more than three-quarter of physicians believe that Hexaxim® usage could increase the parents’ compliance to the immunization program, which is a much higher percentage compared with the survey carried out in the United States in 2008 in which only 26.0% of physicians agreed that a combination vaccine would increase the parents’ compliance [30]. This inconsistency is based upon the Malaysian physicians’ previous experience with Pentaxim® vaccine where they noticed an increase in parents’ compliance to the NIP, compared with monovalent vaccines (Hep B or oral polio vaccine) used earlier. The current study findings are in line with the findings of Kalies et al., i.e., the physicians’ perceptions toward parents’ better compliance if Hexaxim® is incorporated in the NIP [33]. Kalies et al. [33] found that combination vaccines were shown to be associated with improved timeliness of vaccination, with the percentage of subjects completing the full immunization series in time increasing with the use of higher valence vaccines. As a result of their positive perception regarding Hexaxim®, more than three-quarter of physicians supported using Hexaxim® in the immunization schedule.

The followings are the limitation of this study: (1) data were collected only from the PHCs of the states of Selangor and the Federal Territory of Kuala Lumpur due to limited budget and short study duration; (2) due to accessibility issues, two PHCs were excluded from the study plan; and (3) systematic sampling of nurses for MCH that have more than five nurses was not employed appropriately in some health centers since some nurses were absent or transferred to other departments on the day of data collection, which forced the researchers to choose a convenient sample.

Based upon the results of this study, the following are the proposed recommendations for the Ministry of Health (MOH): (1) Hexaxim® vaccine can be adopted by the MOH and replace Pentaxim® and Hep B starting with the next birth cohort; (2) an evaluation study should be conducted to explore the satisfaction level of parents, nurses, and physicians after Hexaxim® vaccine usage; (3) the immunization schedule can be modified to exclude extra routine visits that does not include vaccination based upon the desire of the parents; (4) any current or future vaccines that need reconstitution can be replaced or not incorporated, since it can lead to handling errors or needlestick injury based upon nurses’ perceptions.

## Conclusion

The results of this study recommended incorporation of Hexaxim® within the Malaysian NIP because the use of Hexaxim® had a lower cost per dose and demonstrated substantial direct and indirect cost savings for healthcare providers and parents/caregivers, compared with Pentaxim® and Hep B. This is supported by the high percentage of positive perceptions regarding Hexaxim® obtained from parents and healthcare providers. In addition, Hexaxim® reduces clinic visits, handling errors, and number of injections, which translate to better acceptability, convenience, and increased compliance.

## Supplementary Information

**Additional file 1.**

## Data Availability

The datasets used and/or analyzed during the current study are available from the corresponding author on reasonable request.

## Abbreviations

DTaP: Diphtheria, tetanus, and acellular pertussis
FIC: Fully immunized child
HCPs: Healthcare professionals
Hib: *Haemophilus influenzae* type b
Hep B: Hepatitis B
IPV: Inactivated polio vaccine
MCH: Maternal and child health
MOH: Ministry of Health
NIP: National Immunization Program
PHCs: Primary healthcare centers
RM: Malaysian ringgit
