# Supplementary material for: Economic impact of switching from partially combined vaccine “Pentaxim® and hepatitis B” to fully combined vaccine “Hexaxim®” in the Malaysian National Immunization Program
Source: BMC Health Serv Res. 2022 Jan 5;22:34. doi: 10.1186/s12913-021-07428-7 (PMC8734051; doi:10.1186/s12913-021-07428-7)
Supplement: Supplementary file 1 — Additional file 1. [file 12913_2021_7428_MOESM1_ESM.docx]

**SUPPLEMENTARY TABLES**

**Supplementary Table 1: Vaccine costing questionnaire (health clinic version)**

| **Serial No.** | **Item** | **Number** | |
| --- | --- | --- | --- |
| 1. | Staff nurse |  | |
| 2. | Community health nurse |  | |
| 3. | Family medicine specialist |  | |
| 4. | Medical Officer |  | |
| 5. | Children vaccinated with hepatitis B (last year) |  | |
| 6. | Children vaccinated with Pentaxim (last year) |  | |
|  | | | |
|  | **Consumables** | **Pentaxim** | **Hepatitis B** |
| 7. | Number of cotton swab used per dose |  |  |
| 8. | Number and volume of syringes used per dose:  a.: Reconstitution syringe  b.: Injection syringe | a.  b. | a.  b. |
| 9. | Number and size of needle used per dose:  a.: Reconstitution needle  b.: Injection needle | a.  b. | a.  b. |
| 10. | Number and volume of vials used per dose |  |  |
| 11. | Safety box volume |  |  |
| 12. | Number of safety boxes used weekly |  |  |
|  | | | |
|  | **Vaccine hazardous waste disposal** | **Pentaxim** | **Hepatitis B** |
| 13. | Number and weight of syringes disposed per dose |  |  |
| 14. | Number and weight of needles disposed per dose |  |  |
| 15. | Number and weight of vials disposed per dose |  |  |
|  | | | |
|  | **Vaccine wastage rate** | **Pentaxim** | **Hepatitis B** |
| 16. | Number of doses per vial |  |  |
| 17. | Number of vials opened for use during the last year |  |  |
| 18. | Number of closed vials discarded during the last year |  |  |
| 19. | Number of doses administered during the last year |  |  |
|  | | | |
|  | **Vaccine cold chain storage** | | |
|  | **(A)** | | |
|  | **Refrigerator type** | **TCW 3000** | **TCW 2000/domestic fridge** |
| 20. | Quantity |  |  |
| 21. | Gross volume (L) |  |  |
| 22. | Vaccine storage capacity (L) |  |  |
| 23. | Freezing gross volume (L) |  |  |
| 24. | Freezer ice pack capacity |  |  |
| 25. | Energy usage per hour (kWh) |  |  |
|  | | | |
|  | **(B)** | | |
|  | **Cold Box** | **For current usage** | **For current stock** |
| 26. | Quantity |  |  |
| 27. | Volume |  |  |
| 28. | Number of ice packs inside cold box |  |  |

**Supplementary Table 2: Vaccine costing questionnaire (district health office version)**

| **Serial No.** | **Item** | **Pentaxim** | **Hepatitis B** |  |
| --- | --- | --- | --- | --- |
| 1 | Price per dose |  |  |  |
| 2 | Number of doses supplied during 2019 |  |  |  |
| 3 | Vaccine coverage rate (%) |  |  |  |
|  | **Item** | | **Price** | |
| 4 | Bag/box of cotton wool swab | |  | |
| 5 | Box of alcohol swabs (if used at your Health Centre) | |  | |
| 6 | Syringe 3 mL | |  | |
| 7 | Needle 25 Ga × 25 mm | |  | |
| 8 | Needle 23 Ga × 25 mm | |  | |
| 9 | Unused safety box (20, 15, 10, and 5 L) | |  | |
| 10 | Disposal of 1 kg of hazardous waste | |  | |
| 11 | Electricity (kWh) | |  | |
|  | **Question** | | **Answer** | |
| 12 | How many pieces of cotton swab are inside each bag/box? | |  | |
| 13 | How many pieces of alcohol swabs are inside each box (if used)? | |  | |
| 14 | How much is the birth cohort for 2019? | |  | |
| 15 | Average annual salary (according to years of experience):  a.: Staff nurse  b.: Community health nurse | | a.:  b.: | |

**Supplementary Table 3: Time and motion chart**

| **A. Nurse’s clinical profile** | |
| --- | --- |
| **Nurse Specialty** | **Staff Nurse Community Health Nurse** |
| **Experience (years)** |  |

| **B. Time and motion data for administrative and child wellbeing tasks** | | |
| --- | --- | --- |
| **Steps** | **Service provider tasks** | **Time (s)** |
| 1. | Registration |  |
| 2. | Interview with parent |  |
| 3. | Anthropometry |  |
| 4. | Physical examination |  |
| 5. | Child development assessment |  |
| 6. | Health education |  |
| 7. | Assigning next appointment |  |
| 8. | Documentation |  |
|  | | |
| **C. Time and motion data for hepatitis B vaccine** | | |
| **Steps** | **Service provider tasks for hepatitis B administration** | **Hepatitis B (s)** |
| 1. | Sterilization of hands |  |
| 2. | Open the cold box |  |
| 3. | Take HepB vial out of cold box |  |
| 4. | Unpack/split and unwrap syringe |  |
| 5. | Unpack/split and unwrap needle |  |
| 6. | Afﬁx needle 1 to syringe |  |
| 7. | Shake vial |  |
| 8. | Uncap vial/expose stopper |  |
| 9. | Uncap needle 1 |  |
| 10. | Plunge needle 1 into the vial |  |
| 11. | Withdraw content into syringe |  |
| 12. | Detach needle 1 from vial |  |
| 13. | Affix needle 2 to syringe |  |
| 14. | Inspect/Calibrate the filled syringe before injection |  |
| 15. | Prepare child in the appropriate injection position |  |
| 16. | Prepare and apply first cotton swab on the child |  |
| 17. | Uncap needle 2 |  |
| 18. | Inject vaccine into the child thigh |  |
| 19. | Prepare and apply second cotton swab on the child |  |
| 20. | Dispose needle, syringe, and vial into safety box |  |
| **Total Time** (s) | |  |
|  | | |
| **D. Time and motion data for Pentaxim (DTaP-IPV/Hib) vaccine** | | |
| **Steps** | **Service provider tasks for Pentaxim administration** | **Pentaxim (s)** |
| 1. | Sterilization of hands |  |
| 2. | Open the cold box |  |
| 3. | Take out Pentaxim out of cold box |  |
| 4. | Unwrapping pack |  |
| 5. | Take out vial and prefilled syringe out of the pack |  |
| 6. | Unpack/split and unwrap extra syringe |  |
| 7. | Unpack/split and unwrap extra two needles |  |
| 8. | Uncap vial/Expose stopper |  |
| 9. | Uncap the needle 1of preﬁlled syringe |  |
| 10. | Transfer content of syringe into vial |  |
| 11. | Detach needle 1 from vial |  |
| 12. | Shake vial to make homogenous solution |  |
| 13. | Dispose of prefilled syringe and needle 1 |  |
| 14. | Affix and uncap needle 2 to syringe 2 |  |
| 15. | Withdraw vial content into syringe 2 |  |
| 16. | Detach needle 2 from syringe 2 |  |
| 17. | Affix needle 3 to syringe 2 |  |
| 18. | Inspect/Calibrate the filled syringe before injection |  |
| 19. | Prepare child in the appropriate injection position |  |
| 20. | Prepare and apply first cotton swab on the child |  |
| 21. | Uncap needle 3 |  |
| 22. | Inject vaccine into the child thigh |  |
| 23. | Prepare and apply second cotton swab on the child |  |
| 24. | Dispose needles, syringe, and vial into safety box |  |
| **Total time** (s) | |  |

Abbreviation: DTaP-IPV/Hib, diphtheria, tetanus, pertussis, poliomyelitis, and Haemophilus influenzae type b.

**Supplementary Table 4: Parents/Caregivers’ questionnaire**

**Section A: Parents/Caregivers’ sociodemographic profile**

**Instructions**: Please tick the appropriate answer in the box and fill when necessary.

**1. Age of the caregiver/parent:** years

**2. Gender**

Male Female

**3. Ethnicity**

Malay Chinese

Indian Other, please specify:……………………______________

**4.** **Relationship with the child**

Mother Father Family member Guardian

**5. Marital status**

Single Married Divorced Widowed

**6. Educational level**

Primary or less Secondary Post-secondary

**7. Occupation**

Public sector Private sector Self-employed

Unemployed Housewife

**8. Age of your child to be vaccinated today:** Days/weeks/months/years

**9. You are attending this clinic today so that your child get:**

DTaP-IPV/Hib vaccine Hepatitis B vaccine Mono Measles

MMR (Measles, Mumps & Rubella) DTaP-IPV/Hib + Hep B

**10. If you are working, how much is your monthly earnings?** RM

**Section B: Parents/Caregivers’ Perceptions regarding employment of six vaccines in one injection in the national immunization program**

**11. One injection that contains six vaccines will reduce my child’s pain compared with two injections that contain the same number of vaccines.**

Strongly agree Agree Neutral

Disagree Strongly disagree

**12. One injection that contains six vaccines will reduce the number of visits I have to make to the vaccination clinic compared with two injections that contain the same number of vaccines.**

Strongly agree Agree Neutral

Disagree Strongly disagree

**13. One injection that contains six vaccines will reduce my transportation expenses compared with two injections that contain the same number of vaccines.**

Strongly agree Agree Neutral

Disagree Strongly disagree

**14. One injection that contains six vaccines will lead to higher compliance with the vaccination schedule compared with two injections that contain the same number of vaccines.**

Strongly agree Agree Neutral

Disagree Strongly disagree

**15. In case there is an alternative vaccine that can reduce the number of injections and visits to the vaccination clinic, I believe that the current immunization schedule needs to be reviewed**:

Strongly agree Agree Neutral

Disagree Strongly disagree

**Section C: Caregivers/Parents’ out of pocket expenses**

**16. Please specify your house address**:

- State: …………………………………………………………………………………..
- District: ………………………………………………………………………………..
- City: ……………………………………………………………………………………
- Postcode: ………………………………………………………………………………
- Street: …………………………………………………………....................................

**Section D: Caregivers/Parents’ loss of productivity**

**17. Did you a take a time off work today to vaccinate your child?**

Yes No

**18. This time off work is considered as:**

A paid time off unpaid time off

**Supplementary Table 5: Healthcare professionals’ questionnaire (nurses’ version)**

**Section A: Nurses’ demographic and clinical practice profile**

**Instructions**: Please tick the appropriate answer in the box and fill when necessary

1. **Age**: years

2. **Gender**:

Male Female

3. **Profession**:

Staff Nurse Community Health Nurse

**4.** **Current role in the vaccination process**:

Only prepare injections Only administer injections

Prepare and administer injections Only prescribes vaccines

Prescribe, prepare, and administer vaccine

**5.** **Years of experience** **in childhood vaccination**:

**Section B: Nurses perception regarding DTaP-IPV/Hib (Pentaxim) vaccine**

**6. I think that DTaP-IPV/Hib vaccine reconstitution is time lost that can be spent with parents/infants:**

Strongly agree Agree Neutral

Disagree Strongly disagree

**7. I think that DTaP-IPV/Hib vaccine reconstitution requires too many steps compared with other vaccines** (such as Hep B)**:**

Strongly agree Agree Neutral

Disagree Strongly disagree

**8.** **I think that** **DTaP-IPV/Hib vaccine reconstitution introduces the risk of handling errors** (such as inadequate shaking of the vial after reconstitution or not aspiring the whole content of the reconstituted vial):

Strongly agree Agree Neutral

Disagree Strongly disagree

**9.** **I think that** **DTaP-IPV/Hib vaccine reconstitution introduces the risk of needle stick injury compared with other vaccines** (such as Hep B).

Strongly agree Agree Neutral

Disagree Strongly disagree

**Section B: Nurses’ perception regarding employment of 6-in-1 injection vaccine in the national immunization program**

**10. I think that replacing 6-in-1 injection instead of DTaP and Hepatitis B vaccines in the national immunization program will reduce the daily work burden borne by nurses.**

Strongly agree Agree Neutral

Disagree Strongly disagree

**11. I think that replacing 6-in-1 injection instead of DTaP and Hepatitis B vaccines in the national immunization program will decrease the daily patient influx to the vaccination facility.**

Strongly agree Agree Neutral

Disagree Strongly disagree

**12. I would be interested in replacing 6-in-1 injection instead of DTaP and Hepatitis B vaccines in the national immunization program.**

Strongly agree Agree Neutral

Disagree Strongly disagree

**Supplementary Table 6: Healthcare professionals’ questionnaire (physicians’ version)**

**Section A: Physicians’ demographic and clinical practice profile**

**Instructions**: Please tick the appropriate answer in the box and fill when necessary.

1. **Age**: Years

2. **Gender**:

Male Female

3. **Profession**:

Family Medicine Specialist Medical Officer

**4.** **Current role in the vaccination process**:

Counselling regarding childhood vaccination

Supervision for the process of childhood vaccination

Both

Others, please specify: ……………………………………………………...

**5.** **Years of experience** **in childhood vaccination**:

**Section B: Physicians’ perception regarding employment of Hexaxim in the national immunization program**

**6. I would be interested in replacing Hexaxim instead of Pentaxim and Hepatitis B vaccines in the national immunization program.**

Strongly agree Agree Neutral

Disagree Strongly disagree

**7. Caregivers/Parents of my pediatric patients would be interested in replacing Hexaxim instead of Pentaxim and Hepatitis B vaccines in the national immunization schedule.**

Strongly agree Agree Neutral

Disagree Strongly disagree

**8. I believe that replacing Hexaxim instead of Pentaxim and Hepatitis B vaccines in the national immunization schedule can result in cost savings to the government.**

Strongly agree Agree Neutral

Disagree Strongly disagree

**9. I believe that replacing Hexaxim instead of Pentaxim and Hepatitis B vaccines in the national immunization schedule can reduce the number of patients attending the vaccination facility on daily basis.**

Strongly agree Agree Neutral

Disagree Strongly disagree

**10. I believe that replacing Hexaxim instead of Pentaxim and Hepatitis B vaccines can ease the incorporation of new vaccines such as PCV to the current immunization schedule.**

Strongly agree Agree Neutral

Disagree Strongly disagree

**11. I believe that replacing Hexaxim instead of Pentaxim and hepatitis B vaccines will lead to more compliance from parents’ side to the immunization schedule.**

Strongly agree Agree Neutral

Disagree Strongly disagree

Abbreviation: PCV, pneumococcal conjugated vaccine.

**Supplementary Table 7: Formulas and inputs used to calculate vaccines cost components**

| **Cost component** | **Formula** | **Inputs** |
| --- | --- | --- |
| Swab | Number of swab used per dose of vaccine × swab price per piece | - Price of cotton swabs pack f 250 balls = 4.18 RM, then the price of one cotton swab = 0.01672 RM - Price of alcohol swabs pack of 200 swabs = 4.06 RM then the price of one alcohol swab = 0.0203 RM - Either two cotton swabs used or one alcohol plus one cotton swab per dose of vaccine |
| Syringe | Number of syringes per dose of vaccine × price of one syringe × syringes wastage factor | - Price of syringe (3 mL) pack of 100 pieces = 29.35 RM, then the price of one syringe = 0.2935 RM - Syringe wastage factor = 1.11 - Pentaxim, hepatitis B, and Hexaxim vaccines consume one syringe per dose |
| Needle | Number of needles per dose of vaccine × price of one needle × needles wastage factor | - Price of needles (23 Ga × 25 mm) pack of 100 pieces = 8.35 RM, then the price of one needle = 0.0835 RM - Needles wastage factor = 1.11 - Pentaxim, hepatitis B, and Hexaxim vaccines consume two needles per dose |
| Safety box | (Syringe volume × number of syringes per dose of vaccine) + (needle volume × number of needles per dose of vaccine) + (vaccine vial volume × number of vials per dose of vaccine) × (price of empty safety box)/actual safety box volume (mL) (2/3 of safety box gross volume) | - Price of safety box gross volume 2.5 L = 0.425 RM - Price of safety box gross volume 5 L = 0.53 RM - Price of safety box gross volume 10 L = 0.8 RM - Price of safety box gross volume 20 L = 1.33 RM - Pentaxim vaccine consumes 9.013268 mL of safety box space per dose - Hepatitis B vaccine consumes 6.013268 mL of safety box space per dose - Hexaxim vaccine consumes 6.013268 mL of safety box space per dose |
| Hazardous waste Disposal | (Empty syringe weight × number of syringes disposed per dose of vaccine) + needle weight × number of needles per dose of vaccine) + (empty vaccine vial weight × number of empty vials per dose of vaccine)/1000 × hazardous waste disposal price per 1000 g | - Price of disposing 1000 g sharp medical waste is 10 RM - Pentaxim hazardous waste weight per dose is 15 g - Hepatitis B hazardous waste weight per dose is 10 g - Hexaxim vaccine hazardous waste per dose is 10 g. |
| Vaccine wastage rate | Purchase price of vaccine per dose × vaccine wastage factor | - According to the World Health Organization vaccine wastage rate of single dose vial is 5% - Pentaxim purchase price per dose = 52.09 RM - Hepatitis B purchase price per dose = 4.292 RM - Hexaxim shadow price baseline (52.09 + 4.292) = 56.382 RM |
| Cold chain:  Refrigerator | Vaccine package volume per dose (mL) × (refrigerator electricity expenditure per day × electricity price per kWh/refrigerator vaccine storage capacity [mL]) | - Price of electricity per kWh = 0.50873 RM - Pentaxim secondary package volume = 154.411 mL - Hepatitis B secondary package volume = 14.53 mL - Hexaxim secondary package volume = 11.3 mL |
| Cold chain: cold box | (Refrigerator or freezer electricity expenditure kWh × electricity price per kWh/freezer gross volume (mL)] × (ice pack total volume per cold box × vaccine volume per dose/cold box volume [mL]) | - Price of electricity per kWh = 0.50873 RM - Pentaxim secondary package volume = 154.411 mL - Hepatitis B vial volume= 3 mL - Hexaxim vial volume = 3 mL - Ice pack volume = 600 mL - Cold box usually contains 6 or 11 ice packs. |
| Administration time | Monthly specific nurse salary (basic salary)/working hours per month/3600 × time (s) to administer one dose of vaccine | - Staff nurse’s basic salary/month = 1493 + 145 RM increase per each year of experience - Community health nurse’s basic salary/month = 1360 + 100 RM increase per each year of experience - Nurses work for 22 days per month and 9 h per day, which means that the working hours per month for them are equal to 198 h. - Administration time per dose of vaccine was calculated by adding the time required to perform administrative, wellbeing tasks, and vaccine preparation and injection time). - Pentaxim vaccine average administration time per dose of vaccine is 3061.58 s (51 min and 1.6 s). - Hepatitis B vaccine average administration time per dose of vaccine is 3041.35 s (50 min and 41.4 s). - Hexaxim vaccine average administration time per dose of vaccine is 3039.24 s (50 min and 39.2 s). |

Abbreviation: RM, Malaysian Ringgit.
